# Lysosomal Exocytosis: The Extracellular Role of an Intracellular Organelle

**DOI:** 10.3390/membranes10120406

**Published:** 2020-12-09

**Authors:** Brunella Tancini, Sandra Buratta, Federica Delo, Krizia Sagini, Elisabetta Chiaradia, Roberto Maria Pellegrino, Carla Emiliani, Lorena Urbanelli

**Affiliations:** 1 Department of Chemistry, Biology and Biotechnology, University of Perugia, Via del Giochetto, 06123 Perugia, Italy; brunella.tancini@unipg.it (B.T.); sandra.buratta@unipg.it (S.B.); federica.delo@studenti.unipg.it (F.D.); krizia.sagini@hotmail.com (K.S.); roberto.pellegrino@unipg.it (R.M.P.); 2 Department of Veterinary Medicine, University of Perugia, Via S. Costanzo 4, 06126 Perugia, Italy; elisabetta.chiaradia@unipg.it; 3 Centro di Eccellenza sui Materiali Innovativi Nanostrutturati (CEMIN), University of Perugia, Via del Giochetto, 06123 Perugia, Italy

**Keywords:** lysosomal exocytosis, lysosomes, cellular clearance, mTOR, TFEB, TRPLML1

## Abstract

Lysosomes are acidic cell compartments containing a large set of hydrolytic enzymes. These lysosomal hydrolases degrade proteins, lipids, polysaccharides, and nucleic acids into their constituents. Materials to be degraded can reach lysosomes either from inside the cell, by autophagy, or from outside the cell, by different forms of endocytosis. In addition to their degradative functions, lysosomes are also able to extracellularly release their contents by lysosomal exocytosis. These organelles move from the perinuclear region along microtubules towards the proximity of the plasma membrane, then the lysosomal and plasma membrane fuse together via a Ca^2+^-dependent process. The fusion of the lysosomal membrane with plasma membrane plays an important role in plasma membrane repair, while the secretion of lysosomal content is relevant for the remodelling of extracellular matrix and release of functional substrates. Lysosomal storage disorders (LSDs) and age-related neurodegenerative disorders, such as Parkinson’s and Alzheimer’s diseases, share as a pathological feature the accumulation of undigested material within organelles of the endolysosomal system. Recent studies suggest that lysosomal exocytosis stimulation may have beneficial effects on the accumulation of these unprocessed aggregates, leading to their extracellular elimination. However, many details of the molecular machinery required for lysosomal exocytosis are only beginning to be unravelled. Here, we are going to review the current literature on molecular mechanisms and biological functions underlying lysosomal exocytosis, to shed light on the potential of lysosomal exocytosis stimulation as a therapeutic approach.

## 1. Introduction

Lysosomes are intracellular organelles surrounded by a single membrane and displaying a round morphology, first discovered by C. De Duve in the early 1950s [1]. From a biochemical point of view, their distinctive feature is a typically acidic luminal pH. The lysosomal pH has been estimated to be at least 100 times more acidic than its cytosolic counterpart, ranging from 4.5 to 5.0. The differential H^+^ concentration is actively maintained by specific vacuolar ATPases (v-ATPases) [2]. Within their lumen, lysosomes contain about 60 hydrolases, which digest every cell macromolecule (proteins, lipids, polysaccharides, and nucleic acids) into their constituents, while lysosomal membrane proteins, such as transporters and ion channels, also contribute to key lysosomal functions [3,4].

**Figure 1:**
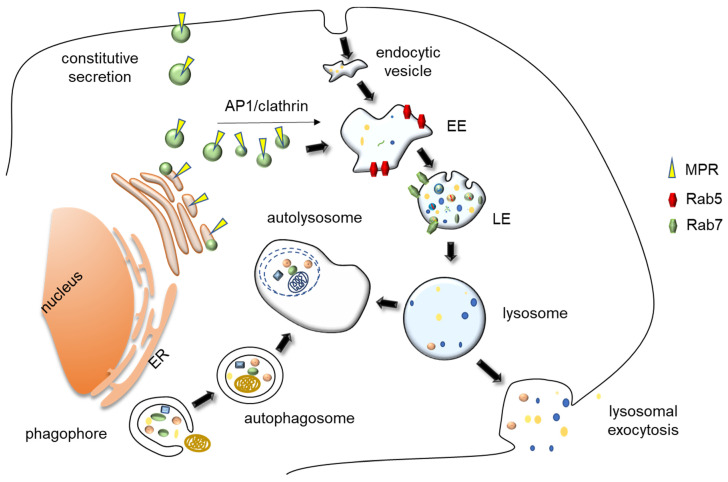
**Representation of lysosomal biogenesis, major trafficking route and exocytosis.** Materials to be degraded into lysosome can be either of extracellular or intracellular origin. Cells receive their extracellular cargoes for degradation by different forms of endocytosis and their intracellular cargoes by different forms of autophagy, namely by macroautophagy, which includes the engulfment of large cytoplasmic entities and damaged organelles within a double membrane structure, the autophagosome. This entity then fuses with lysosomes, forming an autolysosome. Lysosome biogenesis requires both the biosynthetic and endocytic pathways. Lysosomes receive their specific soluble hydrolases and membrane proteins cargos from the “conventional” secretory pathway. Briefly, in the trans-Golgi, M6P receptors bind to proteins carrying mannose 6-phosphate residues, interact with AP1/clathrin complexes, and bud from the apparatus as vesicles. Lysosomal membrane proteins are delivered to lysosomes in a M6P receptor-independent manner, as their transport from the trans Golgi network to the lysosome occurs both by a direct route or indirectly via vesicle fusion with plasma membrane, followed by endocytosis. These vesicles fuse with endosomes, delivering their lysosomal protein precursor content. EE, Early endosomes; LE/MVB, Late endosomes/MultiVesicular Bodies; MPR, Mannose Phosphate Receptors; ER, Endoplasmic Reticulum.

Macromolecules are delivered into lysosomes either extracellularly, via endocytosis or phagocytosis followed by endosome fusion with lysosomes to produce endolysosomes, or intracellularly, via autophagy followed by autophagosome fusion with lysosomes, to form autolysosomes (Figure 1). Once macromolecular substrates are digested within lysosomes, their constituents are exported into the cytoplasm via specific membrane transporters and used either as an energy source, by catabolic reactions, or as building blocks, for anabolic reactions. Therefore, lysosomes, which have long been considered just “waste disposal bags”, are now more correctly regarded as metabolic hubs, whose degradative function is finally regulated by specific nutrient sensing mechanisms, according to cell needs [5]. Moreover, lysosomes also play an important homeostatic role for the cell, because the autophagic process is the only one capable to engulf and then degrade damaged organelles, such as mitochondria and peroxisomes, a key function to maintain cell fitness [6,7].

Finally, lysosomes not only carry out intracellular functions, but are also capable to release their content extracellularly, via lysosomal exocytosis. This process fulfils two main purposes, the repair and/or remodelling of the plasma membrane and the secretion of lysosomal hydrolases and/or functional substrates. To carry out these tasks, lysosomes move close to the plasma membrane and fuse with it in response to specific stimuli. The regulation of this movement by interaction with the cytoskeleton plays a fundamental role in lysosomal exocytosis. In this manuscript, we are going to review the current knowledge on lysosomal exocytosis, describe the underlying molecular mechanisms, and focus our attention on the recent findings concerning the role of lysosomal positioning and movement in lysosomal exocytosis. The pathological implication of lysosomal exocytosis and the targeting of this process for therapeutic purpose will be also discussed.

## 2. Lysosome Biology

### 2.1. Lysosomal Heterogeneity and Lysosomes Related Organelles

The initial characterization of lysosomes as small organelles with a scattered cytoplasmic localization has recently given way to a more complex picture. Lysosomes have an heterogenous size ranging from 100 to 1000 nm, a round morphology and are present in many copies (up to several hundreds), preferentially located in the perinuclear region [8]. Their size and morphology may vary consistently according to the cell type [9,10]. Lysosomal compartment is not only heterogenous, but also highly dynamic. In vivo microscopy has shown that the lysosomal pool actively moves along microtubules from the perinuclear region towards the plasma membrane, by the so-called anterograde transport, and from the proximity of the plasma membrane toward the perinuclear region, by the so-called retrograde transport [11]. Lysosomal pools located in the perinuclear region and in the proximity of the plasma membrane are functionally different: those located in the perinuclear region are characterized by a higher acidity (pH 4.5–5.5) and degradative capacity, whereas those located near the plasma membrane have a higher pH and a lower degradative capacity [12]. Furthermore, endolysosomes are the principal sites of intracellular acid hydrolases activity and are distinct from terminal lysosomes, which act as a store of acid hydrolases [13]. Perinuclear localization appears to be a site for the efficient maturation of endosomes, as the bulk of lysosome-endosome fusion occurs in this region and it may be coupled to their retrograde transport. More peripheral lysosomes appear to be involved mostly in membrane repair and secretion [10,12].

To further complicate this heterogenous picture, lysosomes not only show great heterogeneity within the same cell as well as among different cell types, but in specialized cell types, also co-exist with the so-called lysosomal related organelles (LRO), which are organelles related to the endolysosomal system having mostly secretory function [14]. LROs include melanosomes in the pigment cells, dense granules in platelets, and lytic granules in cytotoxic T lymphocytes [15]. They were initially considered specialized lysosomes destined for secretion, in contraposition with conventional lysosomes regarded as the “final destination” of the degradative pathway. However, it later emerged that conventional lysosomes also possess the machinery for regulated lysosomal exocytosis [16].

### 2.2. Biogenesis and Reformation of Lysosomes

Lysosome biogenesis requires both the biosynthetic and endocytic pathways. Lysosomes receive most of their specific soluble hydrolases, such as proteases, lipases, glycosidases and nucleases, and membrane proteins from the “conventional” secretory pathway. As a matter of fact, the lysosomal enzyme precursor possesses an N-terminal peptide leading to their translation into ER-associated ribosomes. From the ER, they are transported into the Golgi apparatus in membrane-bound vesicles. In the cis-Golgi, they are modified by addition of mannose-6-phosphate (M6P). In the trans-Golgi, M6P receptors bind to proteins carrying M6P residues and bud from the apparatus as clathrin-coated vesicles (CCVs). The assembly of CCV requires the Golgi-localized, gamma adaptin ear-containing, ADP-ribosylation factor-binding (GGA) proteins and the adaptor protein complex 1 (AP1), both binding clathrin and M6P receptor [17]. These vesicles fuse with early endosomes, delivering their lysosomal protein precursor content [18]. At the late endosome pH, which is around 6.0, M6P residues detach from their receptors. Receptors are recycled back into the trans-Golgi, whereas late endosomes fuse with lysosomes, releasing their content of hydrolases, which complete their maturation at the lysosomal acidic pH [19]. A few lysosomal hydrolases are delivered to lysosomes in a manner that is independent from the addition of M6P residues [20]. The most extensively investigated of these hydrolases is β-glucocerebrosidase, whose targeting to lysosomes is mediated by the lysosomal integral membrane protein-2 (LIMP2) [21]. Furthermore, newly synthesized lysosomal integral membrane proteins are delivered to lysosomes by in a M6P-receptor independent manner, as their sorting from the trans Golgi network to the lysosome may occur both by a direct route or an indirect route, i.e., by vesicular transport to the plasma membrane followed by endocytosis [18]. Endocytosis is a complex process, driven by different mechanisms, that commonly begins at the plasma membrane, with its inward invagination leading to the formation of endocytic vesicles. These vesicles fuse with early endosomes, that later mature into late endosomes, characterized by a more acidic pH [22]. Early endosomes are characterized by their association with Rab5 GTPase. When they mature into late endosomes, Rab7 replace Rab5. Subsequently, late endosomes fuse with lysosomes, forming the so-called endolysosomes [23]. Lysosomes may be then reformed from endolysosomes via a fission mechanism. Briefly, the vesicle forming machinery associates with lysosomes to form vesicles and/or tubules that undergo fission. The vesicle forming machinery is made up of adaptor protein complexes, responsible for cargo sorting, coat proteins, such as clathrin, responsible for membrane deformation, and scission proteins, such as dynamin, responsible for membrane fission [24].

### 2.3. Lysosomes as Terminal Degradative Organelles

The fundamental function of lysosomes is degradative. Cargos to be degraded into lysosome can be either of extracellular or intracellular origin. Cells receive their extracellular cargoes for degradation by different forms of endocytosis. These include phagocytosis, which involves the uptake of solid material and is mostly associated to specific cells of the immune system, such as macrophages and dendritic cells, pinocytosis, which involves the uptake of fluids, including the small particles dissolved therein, and receptor-mediated endocytosis, a process driven by ligand–receptor interaction on the plasma membrane [25]. On the other hand, lysosomes receive their intracellular cargoes by different forms of autophagy (“self-eating”). Autophagy is a degradative process of pivotal importance for cell homeostasis. It is defined by two main features, i.e., the specific degradation of cytoplasmic material or damaged organelles, and the involvement of the lysosome as a terminal degradative compartment [6]. There are different types of autophagy, differing in the modality of cargo selection: macroautophagy (often simply called autophagy), microautophagy, and chaperone-mediated autophagy (CMA). In the case of CMA, membrane fusion is not involved. The cargo is specifically represented by cytosolic proteins bearing a KFERQ-like motif that form a complex with the co-cytosolic chaperone HSC70. This complex is then delivered within lysosomes via direct translocation across the lysosomal membrane, upon interaction with a specific receptor, the 2A isoform of the LAMP2 protein [26]. During the microautophagic process, cytoplasmic protein complexes interacts with the lysosomal membrane and are delivered within the organelle by membrane invagination [27]. Although this process involves cytosolic proteins bearing a KFERQ-like motif interacting with the co-chaperone HSC70, it does not involve LAMP2A and implicates membrane invagination [28]. As for macroautophagy, it is by far the best characterized process. Its main feature is the engulfment of large cytoplasmic entities and/or damaged organelles within a double membrane structure, the autophagosome, which then fuse with lysosomes to form an autolysosome, using a molecular machinery which resembles that responsible for the fusion of late endosomes with lysosomes [29].

### 2.4. Lysosomes as Signaling Hub

The autophagic mechanism and the lysosomal degradative processes are finely tuned. In fact, lysosomes must sense the metabolic status of the cell to coordinate cargo degradation and cell metabolic needs, and to remove damaged material, thus maintaining cell homeostasis. In particular, lysosomal membrane plays a pivotal role in coordinating catabolic and anabolic cell reactions [30,31]. The two main protein complexes which are responsible for the transduction of anabolic and catabolic signaling arising from lysosomes are the mechanistic target of rapamycin complex 1 (mTORC1), which is a master regulator of cell growth and metabolism, and the AMP-activated protein kinase (AMPK), which is the major cellular sensor of energy stress [32]. 

mTOR Complex 1 (mTORC1) is made of three main components: mTOR itself, the regulatory-associated protein of mTOR (Raptor) and the mammalian lethal with SEC13 protein 8 (MLST8) [33]. mTORC1 is activated only in the presence of both growth factors and nutrients, by different signaling cascades, which have been extensively reviewed elsewhere [34]. When nutrients are abundant, mTOR is recruited to the lysosomal membrane and the kinase activity is activated. On the other hand, when nutrients are scarce, mTOR is inactivated and released from the lysosomal surface [35]. The mTOR recruitment on the lysosomal surface depends on a specific complex, which is named LYNUS (lysosomal nutrients sensing) machinery [36]. It consists of the v-ATPase complex, the transmembrane protein SLC38A9, the pentameric Ragulator complex, whose subunits are known as LAMTOR 1 to 5 (late endosomal/lysosomal adaptor, MAPK and mTOR activator 1) and a heterodimeric GTPase complex [37]. The v-ATPase is the protonic pump which is responsible for the maintenance of the acidic lysosomal lumen, while SLC38A9 is both an amino acid transporter and a sensor for arginine [38]. The heterodimeric GTPase complex has a key role: in nutrient abundance condition, it recruits mTORC1 on the lysosomal surface by interaction with another GTPase, Rheb, which is also localized on the lysosomal membrane. The Rag GTPases are of for different types (A, B, C and D) and the heterodimer is composed of either A or B, which appear to be functionally equivalent, in association with either C or D [39,40]. The recruitment is mediated by the GEF (guanine Exchange factor) activity of the Ragulator complex, which mediates the loading of GTP on RagA/B. Once activated, Rag GTPases recruit mTOR to lysosomes. The interaction with Rheb complete the activation of the kinase activity of mTOR. On the other hand, when the nutrient availability is poor (the system has been mostly characterized in condition of aminoacid or cholesterol depletion) mTOR is inactivated, due to a stronger interaction among Ragulator, Rags and the rest of the LYNUS complex [41].

While mTOR is sensitive to the abundance of nutrients such as amino acids, another important complex, AMPK, is instead sensitive to glucose availability. Again, the Ragulator platform plays a key role in the sensing mechanism. When glucose is abundant and glycolysis runs steady, its intermediate fructose-1,6-bisphosphate (FBP) interacts with aldolase (the glycolytic enzyme that is responsible for breaking down FBP into dihydroxyacetone phosphate and glyceraldehyde phosphate). Upon FBP binding, aldolase binds to v-ATPase on the lysosomal surface. Conversely, in the absence of glucose FBP is not sufficient to ensure the aldolase/v-ATPase interaction. In this condition, the interaction among v-ATPase, Ragulator and Axin is favoured, leading to LKB1 (liver kinase B1) and AMPK recruitment, then to AMPK activation by phosphorylation. At the same time, interaction with Axin arrests Ragulator GEF activity toward Rag, inducing mTOR dissociation and inactivation. Interestingly, the Ragulator activity is not sensitive only to glucose starvation, as a similar behaviour was observed during lysosomal deacidification induced by lysosomal stressors [42,43].

These two main pathways converging their signals on the lysosomal membrane have several downstream effectors that regulate cell metabolism. Regarding lysosomes, the best known of them is the transcription factor EB (TFEB) [30]. When nutrients are abundant, mTORC1 associates to the lysosomal membrane, phosphorylates TFEB and retains it on the lysosomal surface, blocking its translocation into the nucleus. Instead, when nutrients are scarce, mTOR is inactivated and TFEB is dephosphorylated. In this condition, TFEB can migrate into the nucleus and promote the transcription of the CLEAR (coordinated lysosomal expression and regulation) gene network [44]. This network includes most lysosomal genes, such as soluble hydrolases and membrane proteins [45], whose expression at high levels is fundamental for lysosomal biogenesis and autophagy.

Nutrient availability is a fundamental signal also for calcium release from cytosolic compartments, including the lysosome. In fact, lysosomes store a significant amount of Ca^2+^ [46], which can be released via two types of channels: transient receptor potential cation channels (TRPMLs) encoded by mucolipin genes, such as mucolipin1 (MCOLN1, also known as TRPML1) and two-pore channels (TPCs), both activated by PI(3,5)P2 (phosphatidylinositol 3,5-bisphosphate) [47,48].

Starvation is known to induce TRPML1-mediated Ca^2+^ efflux [49]. The peri-lysosomal increase of Ca^2+^ activates calcineurin, a calcium/calmodulin dependent protein phosphatase, which also de-phosphorylates TFEB, further reinforcing the starvation signal inducing the nuclear translocation of TFEB. This translocation improves lysosomal biogenesis and autophagy, implements the lysosomal degradation of unnecessary intracellular material, prompts nutrient recycling, and provides building blocks to satisfy cell needs [50]. TRPML1 also regulates mTORC1 through activating calmodulin, and both TRPML1 and calmodulin are required for mTORC1 reactivation during prolonged starvation [51]. Ca^2+^ release is important for lysosomal transport and positioning, as its release induces the retrograde transport of lysosomes versus the perinuclear region via interaction with dynein motor. Lysosomes located in the perinuclear region have a more acidic pH, which usually corresponds to a higher degradative capacity. The perinuclear localization also favours lysosome fusion with autophagosome during the autophagic process [52]. 

## 3. Lysosomal Exocytosis

**Figure 2:**
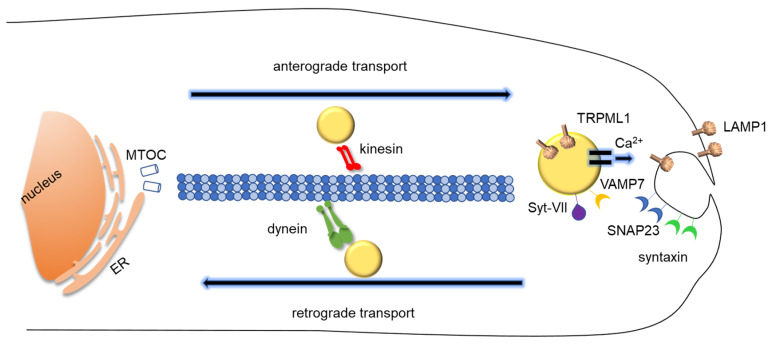
**Schematic representation of lysosomal exocytosis.** Lysosomes migrates from the perinuclear region to plasma membrane proximity and fuse with the plasma membrane, releasing their content extracellularly. Most lysosomes are localized around the nucleus, but a pre-requisite for lysosomal exocytosis is their transport in the proximity of the plasma membrane along microtubules via kinesin motor proteins. The dynamics of lysosomes within cytoplasm also includes the retrograde transport from periphery to nucleus along microtubules, mediated by dynein motor proteins. Vesicle-associated membrane protein 7 (VAMP7) is present on the surface of lysosomes and interacts with syntaxin-4 and with synaptosome-associated protein of 23 kDa (SNAP23) on the plasma membrane. The lysosomal Ca^2+^ channel TRPML1 provides Ca^2+^ for lysosomal membrane fusion, which is sensed by Syt-VII. ER, Endoplasmic Reticulum, MTOC, microtubule-organizing center.

The term lysosomal exocytosis refers to the regulated extracellular release of lysosomal enzymes. During this process, lysosomes migrate from the perinuclear region to the cell surface proximity and fuse with the plasma membrane, releasing their contents extracellularly [53,54] (Figure 2). It is a Ca^2+^-regulated process that has an important role in secretion and plasma membrane repair. Therefore, it plays a pivotal role in several physiological process, such as bone resorption and antigen presentation. Lysosomal exocytosis was initially assessed by detecting lysosomal enzymes activity into cell culture medium, whereas currently the most frequently used assay is the detection of lysosomal membrane proteins such as LAMP1 or even TRPML1 on the plasma membrane [55,56,57]. In fact, the extracellular presence of lysosomal enzymes may be due to another cellular pathway. As a matter of fact, a certain number of vesicles may escape the conventional secretory route, fuse with plasma membrane, and release their content extracellularly [58]. Lysosomal hydrolases released in the extracellular environment may still reach the lysosome. In fact, these proteins are recaptured by M6P receptors localized on at the plasma membrane, stimulating their internalization by the endocytic process, followed by their delivery to lysosomes [59]. Lysosomal exocytosis is considered as an “unconventional secretion” process implicating the direct fusion of lysosomes with plasma membrane [53]. 

### 3.1. The Molecular Machinery of Lysosomal Exocytosis

#### 3.1.1. Lysosomal Movement and Positioning: How Lysosomes Translocate During Lysosomal Exocytosis

Most lysosomes are localized around the nucleus and the microtubule-organizing centre (MTOC) [11], but a pre-requisite for lysosomal exocytosis is their transport in the proximity of the plasma membrane [60]. The lysosome anterograde transport along microtubules is relatively fast (about 0.5 µm s^−1^) and comparable to other types of vesicle transport [61]. It covers rather long distances and is affected by lysosome size [62]. As for other types of vesicle transport, it requires the interaction among motor proteins, regulators, and adaptors [63], although specific components such as BORC (BLOC-one-related complex, where BLOC stands for biogenesis of lysosome-related organelles complex) are required for the transport of lysosomes, but not of synaptic vesicle precursors in mammalian axons [64].

Kinesin superfamily proteins (KIFs) are a large family of motor proteins involved in nucleus to periphery transport along microtubules. They are typically made up of a motor domain binding to microtubules and a tail domain linking specific adaptors or cargos [65]. Several kinesins affect lysosome movement, including kinesin-1, -2, and 3 [66,67,68], but kinesin-1 is the best-characterized. Like most kinesins, it comprises two heavy chains (KIF5A, KIF5B, or KIF5C) and two light chains (KLC1, KLC2, KLC3, or KLC4) [69]. Several studies have shown that lysosomes and late endosomes bind to a few kinesins, but there is no clear correspondence of one organelle to one kinesin. So far, only the kinesin-1 heavy chain KIF5B has been identified as exclusively associated with lysosomes [70].

Small GTPases have an important role in regulating vesicle transport. Arl8 has been discovered as the primary regulatory GTPase on the lysosome. It is a member of the Arf family and two Arl8 genes have been identified Arl8a and Arl8b [71]. Among 30 members of this family, only Arl8 has been found to be associated, although not exclusively, with lysosomes [72]. Arl8 links lysosomes to kinesin-1 via the large protein complex called BORC, which comprises 8 subunits, from BORCS1 to BORCS3 in common with the BLOC-1 complex involved in the biogenesis of LROs [73], and from BORCS4 to BORCS8 specific for lysosomes [52,74]. The association of BORC complex with lysosomes occurs on the cytoplasmic side of the organelle.

Once bound to the lysosomal membrane in its active form, Arl8 recruits a few factors, namely the adaptors SifA (Salmonella-Induced Filaments A) and Kinesin-Interacting Protein (SKIP), also known as Pleckstrin Homology Domain-Containing Family M Member 2 (PLEKHM2), a protein that then recruits kinesin-1 to lysosomal membranes, and HOPS (homotypic fusion and protein sorting), a complex that is necessary for fusion between late endosomes and lysosomes through Rab7 binding [75]. Membrane phospholipids are also involved in mediating the binding of the lysosomes to kinesins. In fact, another adaptor able to recruit kinesin-1 to lysosomes, FYVE- and coiled-coil-domain-containing protein (FYCO1), has been shown to rely on the ER-anchored protein protrudin to interact with lysosomes. Protrudin binds simultaneously to Rab7 and PI(3)P (phosphatidylinositol 3-phosphate) present on the lysosomal membrane, then transfers the organelle to FYCO1 and kinesin-1 for their movement towards the plasma membrane [76,77].

The dynamics of lysosomes within cytoplasm also includes the retrograde transport from periphery to nucleus along microtubules, mediated by dynein motor protein. The interaction of lysosomes with dynein is usually mediated by Rab7, the small GTPase considered a marker of the late endosomal compartment. Rab7 binds to late endosomes and lysosomes via a specific effector, RILP (Rab interacting lysosomal protein), that in turn interacts with dynein and with a subunit of the dynactin complex, p150^Glued^ [78]. In addition to Rab7-RILP, other complexes have been shown to connect lysosomes to dynein. For instance, the transmembrane protein TMEM55B, also known as phosphatidylinositol-4,5-bisphosphate 4-phosphatase (PIP4P1) binds to the dynein adaptor C-Jun-amino-terminal kinase-interacting protein) (JIP4) to facilitate lysosome transport toward the perinuclear region [79]. As in the case of kinesin interaction, lysosomal membrane phospholipids may be relevant for an interaction with motor proteins. As mentioned above, the TRPML1 Ca^2+^ channel is activated by PI(3,5)P2 on lysosomal membrane. The consequent Ca^2+^ efflux activates the Ca^2+^ sensor apoptosis-linked gene 2 (ALG2), an EF-hand-containing protein which in turn binds to dynein [80].

Microtubules are fundamental for long range transportation of lysosomes, either anterograde or retrograde, but lysosomes also make short range movement interacting with actin cytoskeleton and myosin motor proteins. Lysosome interaction with actin cytoskeleton may have important implications for the maintenance of lysosomal position in the proximity of the plasma membrane, a pre-requisite for lysosomal exocytosis. The Wiskott–Aldrich syndrome protein and scar homolog (WASH) complex has a critical role in mediating the interaction of lysosomes with actin cytoskeleton via Arp2/3 complex [81,82]. Moreover, the WASH complex interacts with a subunit of BLOC-1 complex, involved in lysosomes–endosomes formation and trafficking. As WASH co-localizes with γ–tubulin, it has been speculated that WASH may have a role in lysosome–endosome transport connecting actin and microtubules [83]. Further, an actin motor protein, the myosin heavy chain IIA (NMHC IIA) has been demonstrated to be involved in the positioning of lysosomes at the periphery of the cell [84]. Interestingly, evidence has been provided that F-actin mesh also acts as a sort of barrier slowing down the movement of vesicles via their interaction with myosin [85].

#### 3.1.2. Docking and Fusion

The mechanism of membrane fusion has been widely investigated, e.g., in autophagosome–late endosome fusion, or in the fusion of late endosomes with lysosomes [86,87]. The specific molecular events underlying lysosomes fusion with plasma membrane are similar, although they have been far less investigated. This process requires the formation of the N-ethylmaleimide-sensitive factor attachment receptor (SNARE) complexes. There are two groups of SNAREs: vesicle-associated or v-SNAREs, and target-associated or t-SNAREs, which are localized on the destination membrane. SNARE proteins are characterized by a coiled-coil domain stretch containing 60–70 amino acids called the SNARE motif [88]. During membrane fusion, SNARE proteins on opposite membranes form a trans-SNARE complex that pulls membrane closes to each other. Usually, the final fusion step is mediated by calcium release [88]. In the case of lysosomes, it has become clear that vesicle-associated membrane protein 7 (VAMP7) is present on the surface of lysosomes and interacts with syntaxin-4 and with synaptosome-associated protein of 23 kDa (SNAP23) on the plasma membrane. Upon this interaction, lysosomes are docked to the plasma membranes, but membranes are still distinct [89]. The final fusion step requires the local release of Ca^2+^. In the case of lysosomal exocytosis, it has now become clear that, as well as the ER, lysosomes are a store of Ca^2+^, with a concentration almost like the ER [90]. Lysosomal Ca^2+^ ions can be released through TRPML1 Ca^2+^ channel, which facilitates the fusion of lysosomal and plasma membranes [91]. To prompt membrane fusion, the local release of Ca^2+^ must be sensed by specific sensors. The best known of them belong to the synaptotagmin (Syt) family, whose members have two Ca^2+^ binding domains on the cytoplasmic side. In the case of lysosomal exocytosis, Syt-VII has been identified as the lysosomal sensor [92]. Once bound to Ca^2+^, Syt-VII may possibly increase phosphoinositide binding with the juxtaposed SNARE complexes and facilitate membrane curvature, prompting lipid bilayer mixing [93]. However, there is evidence that other Ca^2+^ sensors may participate in lysosomal exocytosis [54,94]. These additional sensors include calmodulin, which is possibly implicated also in late endosome–lysosome fusion [95], and ALG2, the lysosomal targeted Ca^2+^ sensor which is also responsible for the interaction with the dynein motor [39].

#### 3.1.3. Regulation of Lysosomal Exocytosis

Lysosomes localized around the cell nucleus must be transported toward cell periphery along microtubules before their fusion with the plasma membrane. Therefore, the regulation of lysosome anterograde transport, peripheral positioning and localized membrane fusion is fundamental to implement lysosomal exocytosis. Conversely, factors favouring retrograde transport and perinuclear positioning are an obstacle for lysosomal exocytosis. In polarized epithelial cells, the compartmentalization of the plasma membrane SNARE syntaxin-4 at the basolateral membranes leads to directional lysosomal exocytosis, as membrane fusion takes place at the basolateral membrane, but not at the apical membrane [96]. In macrophages, during phagocytosis, lysosomal exocytosis occurs locally at the site of particle uptake [56].

The maintenance of lysosome position in the proximity of the plasma membrane is important for the regulation of lysosomal exocytosis and strongly depends on the LYNUS status [97]. Nutrient depletion suppresses mTORC1 activity and increases TFEB nuclear localization, leading to a more perinuclear lysosome localization and implementing lysosome biogenesis, allowing for increased lysosomal fusion and autophagy. The nutrient-dependent distribution of lysosomes in the proximity of the perinuclear region involves the BORC complex [98]. Specifically, the BORCS6 subunit, also known as lyspersin, is required to connect the BORC complex to a subunit of the Ragulator complex on the lysosome surface [99]. Nutrient availability may modulate lysosomal exocytosis not only acting on lysosomal movement and positioning, but also by regulating lysosomal Ca^2+^ release, which is essential for fusion with plasma membrane during lysosomal exocytosis. Indeed, Ca^2+^ release is regulated via TFEB, which increases the transcriptional activation of the lysosomal Ca^2+^ channel TRPML1, thus inducing the fusion of lysosomes with the plasma membrane [55]. In turn, to increase its transcriptional activity on lysosomal genes, TFEB must be dephosphorylated. Upon its dephosphorylation, it translocates into the nucleus. TFEB dephosphorylation requires mTORC1 inactivation, which is associated with nutrients availability decrease [29]. Therefore, TFEB on one side implements the degradative ability of lysosomes clustered on the perinuclear region by increasing lysosomal hydrolases expression, but on the other side promotes lysosomal exocytosis by inducing localized release of lysosomal Ca^2+^.

From a general point of view, lysosomal lipids are important regulators of lysosomal membrane trafficking and fusion. Cholesterol and sphingolipids specifically affect lysosome membrane fusion machinery. For instance, the intralysosomal cholesterol level is a factor prompting lysosomal movement from the cell periphery to nucleus and therefore hampering lysosomal exocytosis, as when cholesterol is abundant, the cholesterol sensor oxysterol-binding protein-related protein 1L (ORP1L) interacts with the Rab7/RILP complex, recruiting the dynein/dynactin complex to the lysosome for retrograde transport [100]. SNAREs, such as plasma membrane syntaxin 1 and SNAP25 or secretory vesicle synaptobrevin 1/2 are associated with cholesterol rich domains on membranes [101]. In pathologies characterized by secondary cholesterol accumulation, such as multiple sulfatase deficiency and mucopolysaccharidosis type III, late endosome-lysosome fusion is impaired by an altered localization of the lysosomal SNARE VAMP7, which is sequestered in cholesterol-enriched regions of endolysosomal membranes with a consequent alteration of sorting and recycling, although the effects on lysosomal exocytosis is unclear [102]. Therefore, lysosomal cholesterol sensing favours retrograde transport, but a cholesterol adequate level is a pre-requisite for lysosome fusion with plasma membrane. As for sphingomyelin, its lysosomal accumulation inhibits TRPML1 channel activity, thus hampering the Ca^2+^ release from lysosomes, which is fundamental for membrane fusion during lysosomal exocytosis [103]. Lysosomal enzymes acting on membrane lipids as substrate have also been reported to regulate lysosomal exocytosis. Neuraminidase 1 (Neu1), which cleaves terminal sialic acid residues from glycoproteins and glycolipids, de-glycosylates LAMP1 lysosomal membrane protein and impairs lysosomal exocytosis [104]. Acid sphingomyelinase is released extracellularly when cells are wounded contributing to the restoration of plasma membrane integrity through conversion of sphingomyelin into ceramide, which generates endocytic vesicles internalizing the lesions and resealing the plasma membrane [105].

The specific composition of the lysosomal membrane affects lysosome contacts with other organelles, in turn influencing lysosomal position, and indirectly, lysosome exocytosis. For instance, contacts with the ER promote lysosome localization to the perinuclear area, that favours lysosomal fusion with other organelles but not with the plasma membrane [52]. Negative regulation of lysosomal exocytosis has been reported to be associated with the expression of RNF167-a, a lysosomal-associated ubiquitin ligase, whose expression has been associated with perinuclear clustering of lysosomes [106].

Cytosolic pH possibly plays a role in regulating lysosomal exocytosis, although early reports are contradictory and further studies are possibly needed to address this point. Early observations reported that cytosolic acidification induces the anterograde movement of lysosomes to the peripheral region, thus facilitating lysosomal exocytosis, while cytosolic alkalinization induces retrograde movement [107,108]. In macrophages, lysosomal alkalinization was reported to strongly stimulate lysosomal exocytosis [109]. Other studies provide evidence that extracellular acidification induces the outward movement of lysosomes and lysosome exocytosis, to maintain cytosolic pH [110]. Furthermore, the TRPML3 channel was demonstrated to sense lysosomal pH alkalinization by pathogens, thus triggering Ca^2+^ efflux and pathogen expulsion [111]. Interestingly, lysosomotropic drugs, which alter lysosomal pH inducing alkalinization, have been shown to prompt lysosomal exocytosis, as evidenced by increased levels of the lysosomal enzyme cathepsin D in the extracellular milieu [112]. Many of these drugs are CADs (Cationic Amphiphilic Drugs), i.e., hydrophobic weak bases that are sequestered and protonated within lysosomes [113]. They induce TFEB-mediated lysosomal biogenesis and translocation of lysosomes from the perinuclear zone towards the plasma membrane, increasing lysosomal enzyme release in cell culture medium. The evidence that in cancer cells these chemotherapeutics highly accumulate in lysosomes via cation-trapping, leading to lysosomal exocytosis, indicates that lysosomal exocytosis may be a component lysosome-mediated cancer multidrug resistance [112]. Indeed, lysosomal exocytosis not only induces the release of anticancer drugs that highly accumulate in lysosomes, decreasing their intracellular concentration, but also prompts the secretion of several lysosomal enzymes, such as cathepsins, whose extracellular activity is associated with malignant processes, including invasion, metastasis, and activation of angiogenesis [114],

Other physiological factors have been demonstrated to regulate lysosomal exocytosis. In dendrites, backpropagation action potential consequent to neuronal stimulation have been shown to elicit Ca^2+^ release from lysosomes, an occurrence stimulating the fusion of lysosomes with the plasma membrane [115]. Increased lysosomal size hampers exocytosis. Large lysosomes in fibroblasts from Chediak-Higashi patients show reduced exocytosis, but when normal size was ameliorated by treatment with a protease inhibitor, lysosomal exocytosis was repristinated [116].

## 4. Lysosomal Exocytosis Associated Function

Two essential functions have been attributed to lysosomal exocytosis, i.e. membrane remodelling and secretion. The first one is involved in many physiological processes, regarding either all cells, such as membrane repair, or specialized cell types, such as neurons for neurite growth or macrophages phagocytosis [117]. The secretion of lysosomes is fundamental for many specialized cell activities, such as antigen presentation in antigen presenting cells (APCs) [118], bone absorption in osteoclasts [119] and transmitter release [120] in neurons. Initially, lysosomal exocytosis was considered to be limited to these specialized secretory cells, but later, a few studies clearly indicated that this process occurs in all cell types [121,122]. 

### 4.1. Membrane Repair and Remodeling

Plasma membrane integrity is essential for cell homeostasis and requires active mechanism of repair. It has been discovered that the repair of plasma membrane requires the localized release of Ca^2+^, which triggers the fusion of lysosomes (and possibly of other vesicles) with plasma membrane to replace the damaged membrane with intracellular vesicles membrane [123]. Besides this fundamental aid to plasma repair, lysosomal exocytosis also allows the extracellular release of lysosomal enzymes, which play a role in promoting the endocytosis of damaged membranes, therefore completing the repair process [124].

In specialized cell types, the remodelling of membrane is essential to carry on specific functions. In many cell types of the immune system cells, lysosomal exocytosis plays a key role. For instance, macrophages are involved in the uptake of large extracellular particles, such as apoptotic dying cells but also microbial invaders. This process involves the elongation of membrane structures, that surround the extracellular particle to engulf [125]. The elongation requires the membrane to rapidly form and elongate, and to achieve this objective, cells exploit the fusion of intracellular membranes coming from endosomal/lysosomal compartment with plasma membrane, a process called focal exocytosis [51]. The fundamental role of lysosomal exocytosis in the phagocytic process was demonstrated using dominant negative forms of VAMP7 or Syt-VII, whose expression reduced large particle uptake [56,92]. A similar defect was also present in TRPML1 KO macrophages, further reinforcing the evidence that local release of Ca^2+^ by lysosomal deposits is relevant for phagocytosis [93]. Lysosomal exocytosis is also involved in host–pathogen interaction. Viruses have been demonstrated to hijack the autophagic secretion and to exploit extracellular vesicles biogenesis mechanisms during their release from infected cells [126,127]. Further, a recent study has provided evidence that β-coronavirus could use lysosomal organelles for cellular egress, thus focusing the attention on lysosomal function as therapeutic target [128].

In the nervous system, neurite outgrowth is a process that requires the rapid elongation of plasma membrane at the neurite tip. This process is mediated by the localized exocytosis of intracellular vesicles from the endolysosomal system [117]. In fact, the inactivation of Syt-VII gene significantly reduces lysosomal exocytosis and neurite outgrowth [129]. Another important process in the nervous system which involves lysosomal exocytosis is myelination in Schwann cells. Indeed, it has been demonstrated that the myelin protein P0 is contained within late endosomes/lysosomes in Schwann cells and that this protein is released upon Ca^2+^-dependent lysosomal exocytosis [130].

In cancer cells, lysosomal exocytosis has been involved in several membrane processes that are peculiar of these cells. Cancer cell invasive behaviour requires the formation of invadopodia that overgrow through the basement membrane. This process relies on the remodelling of the extracellular matrix and involves lysosomal exocytosis. This allows not only to release the enzymes required for matrix remodelling, but also to form the membrane protrusion to invade tissue [131]. Indeed, during anchor cell invasion in *C. elegans*, localized lysosomal exocytosis directed by netrin receptor provides a local lysosome membrane source necessary to build up the large protrusion that promotes tissue invasion [132]. Cancer cells are characterized by the so-called pH inversion, i.e., they have a higher intracellular pH and a lower extracellular pH as compared to their normal counterparts. Extracellular acidosis was found to increase the localization of LAMP2 on the plasma membrane, a clear indication of increased lysosomal exocytosis. This finding suggests that cells chronically exposed to an acidic extracellular milieu stimulate lysosomal exocytosis to protect their plasma membrane from damage due to acidic hydrolysis [133]. In addition, there is evidence that lysosomal exocytosis contributes to the maintenance of a relatively high intracellular pH. In fact, lysosomal exocytosis not only allows cells to extrude H^+^ contained in the lysosomal lumen, but also translocates v-ATPases from lysosomal to plasma membrane, where they continue to extrude H^+^ from cytosol to the extracellular environment [134].

### 4.2. Lysosome Secretion

The secretion of lysosomal content, namely lysosomal enzymes, has been indicated to have a different function, either common to many cell types or cell type specific. In osteoclasts, specific proteases and a low pH are necessary for the erosion of the small cavities within the bone matrix. Therefore, osteoclasts rely on lysosomal exocytosis for bone resorption and mutations in genes encoding for lysosomal proteins are the cause of several forms of human osteopetrosis [135,136]. In immune cells, lysosomal exocytosis is fundamental. First, although the extracellular pH is not their optimal one, the extracellular release of lysosomal hydrolases digests pathogens defending the organism against them [111,137]. Moreover, in macrophages, polarized lysosomal exocytosis is important for antigen presentation mediated by MHC class II complex [138]. In B-cells, the formation of an immune synapse, which is required for efficient antigen presentation, involves lysosomal exocytosis, as demonstrated by the role of the lysosomal SNARE VAMP7 [109].

Recent evidence has been provided that, in the CNS, lysosomal exocytosis plays a key role in ATP secretion [120,139]. Lysosomes contain high levels of ATP [139] and P2X4 receptor forms functional ATP-activated cation channels on lysosomal membranes regulated by luminal pH [140]. In astrocytes, ATP release is thought to be dependent on lysosomal exocytosis [141]. In addition, evidence has been provided that, in Schwann cells, HIV-1 gp120 induces lysosomal exocytosis, increasing the secretion of ATP into the extracellular medium [142].

Secretion via lysosomal exocytosis is particularly important for cancer cells. Cancer cells exploit the extracellular release of lysosomal enzymes to degrade the extracellular matrix (ECM) and favour the invasion of adjacent tissue, to propagate invasive signals and to purge lysosomotropic chemotherapeutics [143]. In sarcomas, cancer cells acquire malignant traits by inducing LAMP1-dependent lysosomal exocytosis. The cleavage of LAMP1 sialic acids by Neu1 limits the extent of lysosomal exocytosis, whereas the downregulation of Neu1 induces the accumulation of oversialylated LAMP1 and exacerbate lysosomal exocytosis [143]. A genetic screen aimed at identifying driver genes for lung cancer metastasis found that TMEM106B protein positively modulates the expression of lysosomal genes and increases lysosomal exocytosis, followed by the release of active lysosomal cathepsins, which are required for cancer cell invasion and metastasis [144]. Lysosomal exocytosis allows cancer cells to get rid of anticancer drugs because the enhanced lysosomal sequestration of chemotherapeutics moves drugs away from their intracellular targets, paving the way for cancer multidrug resistance. However, little is known regarding the fate of lysosome-sequestered drugs [112]. A few endolysosomal secreted proteins have been indicated to be important in ECM degradation, such as matrix metalloproteinases and possibly integrins, although lysosomal exocytosis is not the only pathway allowing them to reach plasma membrane [145].

There is also evidence that lysosomal exocytosis is important for the maintenance of cell homeostasis, namely the extracellular elimination of metal ions. In fact, lysosomal exocytosis is fundamental to maintain copper homeostasis. Wilson disease is a disorder due to mutations in the copper transporter ATP7B. This channel is localized into Golgi and pumps copper from the cytosol for protein biosynthesis. When cytosolic copper concentration is too elevated, ATP7B moves from the Golgi to lysosomes and imports it into their lumen, using lysosomal exocytosis to eliminate intracellular copper and release it extracellularly [146]. Similarly, it has been reported that lysosomal exocytosis plays a key role in Zn^2+^ detoxication pathway. Lysosomes serve as Zn^2+^ depots that import Zn^2+^ from cytoplasm when its cytosolic concentration is too high, then release Zn^2+^ outside the cell by exocytosis, alleviating the detrimental effects of its accumulation [147].

Recent results also demonstrated that upon lipophagy, i.e., the specialized form of macroautophagy which is responsible for the degradation of lipid droplets within lysosomes, fatty acids are delivered extracellularly via lysosomal exocytosis [148]. As these fatty acids are presumably taken up by neighbouring cells, this may represent a mechanism of lipid exchange among cells, for signaling and energy purposes.

### 4.3. Lysosomal Exocytosis as Therapeutic Target

Many pathological conditions are characterized by intracellular accumulation of undigested substrates within organelles of the endolysosomal system. Among them, LSDs are a group of inherited metabolic disorders due to mutation either in lysosomal hydrolases or membrane protein and transporters. Due to these deficiencies, lysosomes accumulate unprocessed substances, leading to multisystemic pathological symptoms depending on the type of primary storage material but very often associated to neurodegeneration. In fact, age-related neurodegenerative diseases, such as Alzheimer’s and Parkinson’s diseases, share with LSDs the accumulation of insoluble protein aggregates within organelles of the endolysosomal system [149]. For these groups of pathologies, the stimulation of lysosomal exocytosis has appeared as an attractive therapeutic strategy, aimed at getting rid of undigested substrates by their extracellular expulsion, therefore preserving intracellular homeostasis [150]. In a murine model of metachromatic leukodystrophy, a lysosomal disorder characterized by the deficiency of arylsulfatase A enzyme, which results in the accumulation of sulfatide, the pathology was shown to be associated with the presence of sulfatide in body fluids, released by Ca^2+^-dependent lysosomal exocytosis [151]. TFEB regulates lysosomal exocytosis via Ca^2+^ release from TRPLM1 channel. Its overexpression in murine models of multiple sulfatase deficiency and mucopolysaccharidoses type IIIA has been shown to promote cellular clearance [55]. Similar results were also obtained for glycogenosis type II (Pompe’s disease) [152]. More recently, the indirect activation of TFEB by mTORC1-independent pathways was also proven to be useful to promote cellular clearance. Akt phosphorylates TFEB and represses its nuclear translocation. The pharmacological inhibition of Akt confirmed the promotion of cellular clearance in cell models from neuronal ceroid lipofuscinosis (Batten disease) [153]. Similarly, c-Abl phosphorylates TFEB and represses its nuclear translocation. c-Abl inhibition activates TFEB and promotes cellular clearance in a Niemann-Pick type C (NPC) models, a neurodegenerative disease characterized by cholesterol accumulation in lysosomes [154]. TRPML agonists such as SF-51 and ML-SA1 were also proven effective to promote cellular clearance in NPC [155,156] and mucolipidosis type IV [156]. As for neurodegenerative diseases, TFEB regulates lysosomal exocytosis of tau and its loss of function exacerbates tau pathology and spreading in disorders characterized by tau accumulation, such as Alzheimer’s disease [157]. In Parkinson’s disease, lysosomal exocytosis is involved in clearing intracellular α-synuclein aggregates. The upregulation of lysosomal exocytosis by increasing lysosomal Ca^2+^ levels with three different TRPML1 agonists, ML-SA1, SF-22, and MK6-83, is sufficient to rescue defective α-synuclein accumulation and secretion in neurons [158]. The activation of TRPML1 by ML-SA1 agonist clears the intracellular accumulation of amyloid beta peptide in preclinical models of HIV infection [159]. Altogether, these results validate the potential of this therapeutic approach for neurodegenerative disorders. However, the effect on neighbouring cells of a large amount of toxic materials released in the extracellular space must be carefully assessed. Recent studies demonstrated that in sialidosis, a lysosomal disorder caused by Neu1 deficiency, the excessive release of vesicles from late endosome multivesicular bodies was identified as the pathogenic pathway linking a lysosomal deficiency to generalized fibrosis [160].

## 5. Conclusions

Lysosomal exocytosis has a recognized functional role in plasma membrane repair and secretion, that has been mostly characterized and investigated in specialized cell types, namely of the nervous and immune systems, but has also been recognized to be relevant to maintain cell homeostasis in all cell types. From a methodological point of view, the evidence of an extracellular increase of lysosomal enzymes activity is not sufficient to claim the evidence of lysosomal exocytosis, but the process requires the fusion of the lysosomal membrane with plasma membrane, with the consequent translocation of proteins typically associated with the lysosomal membrane onto the plasma membrane. However, many details of the process are still unknown. Considering the high heterogeneity of lysosomal population, the percentage of total lysosomes that can undergo exocytosis without stimulation, or upon specific stimuli in different cell types is unknown. Several proteins are involved in lysosome transport, positioning, and docking/fusion with the plasma membrane, such as motor proteins, Rabs, and SNAREs. However, these protein families are vast and redundant, and their specific association with the different organelles of the endolysosomal system is only partially known. Lysosomal exocytosis and its most known effectors, such as TFEB andTRPML1, have gained considerable interest as a therapeutic target for a large group of pathologies characterized by the intracellular accumulation of undigested substrates, predominantly protein aggregates, namely LSDs and age-related neurodegenerative disorders. The impact on the surrounding tissue of this “forced clearance” has been limitedly investigated, but the evidence that it may be detrimental for the surrounding tissue underlines the necessity of further elucidation of the molecular basis of lysosomal exocytosis.

## References

[1] De Duve C., Pressman B.C., Gianetto R., Wattiaux R., Appelmans F. (1955). Intracellular distribution patterns of enzymes in rat-liver tissue. Biochem. J..

[2] Saftig P., Klumpermann J. (2009). Lysosome biogenesis and lysosomal membrane proteins: Trafficking meets function. Nat. Rev. Mol. Cell Biol..

[3] Brozzi A., Urbanelli L., Germain P.L., Magini A., Emiliani C. (2013). hLGDB: A database of human lysosomal genes and their regulation. Database.

[4] Xu H., Ren D. (2015). Lysosomal Physiology. Annu. Rev. Physiol..

[5] Lamming D.W., Bar-Peled L. (2019). Lysosome: The metabolic signaling hub. Traffic.

[6] Galluzzi L., Baehrecke E.H., Ballabio A., Boya P., Bravo-San Pedro J.M., Cecconi F., Choi A.M., Chu C.T., Codogno P., Colombo M.I. (2017). Molecular definitions of autophagy and related processes. EMBO J..

[7] Anding A.L., Baehrecke E.H. (2017). Cleaning House: Selective Autophagy of Organelles. Dev. Cell.

[8] Kornfeld S., Mellman I. (1989). The biogenesis of lysosomes. Annu. Rev Cell Biol..

[9] De Araujo M.E.G., Liebscher G., Hess M.W., Huber L.A. (2020). Lysosomal size matters. Traffic.

[10] Oyarzún J.E., Lagos J., Vázquez M.C., Valls C., De la Fuente C., Yuseff M.I., Alvarez A.R., Zanlungo S. (2019). Lysosome motility and distribution: Relevance in health and disease. BBA Mol. Basis Dis..

[11] Cabukusta B., Neefjes J. (2018). Mechanisms of lysosomal positioning and movement. Traffic.

[12] Johnson D.E., Ostrowski P., Jaumouillé V., Grinstein S. (2016). The position of lysosomes within the cell determines their luminal pH. J. Cell Biol..

[13] Bright N.A., Davis L.J., Luzio J.P. (2016). Endolysosomes are the principal intracellular sites of acid hydrolase activity. Curr. Biol..

[14] Delevoye C., Marks M.S., Raposo G. (2019). Lysosome-related organelles as functional adaptations of the endolysosomal system. Curr. Opin. Cell Biol..

[15] Bowman S.L., Bi-Karchin J., Le L., Marks M.S. (2019). The road to LROs: Insights into lysosome-related organelles from Hermansky-Pudlak syndrome and other rare diseases. Traffic.

[16] Andrews N.W. (2000). Regulated secretion of conventional lysosomes. Trends Cell Biol..

[17] Braulke T., Bonifacino J.S. (2009). Sorting of lysosomal proteins. Biochim. Biophys. Acta.

[18] Luzio J.P., Hackmann Y., Dieckmann N.M.G., Griffiths G.M. (2014). The Biogenesis of Lysosomes and Lysosome-Related Organelles. Cold Spring Harb. Perspect. Biol..

[19] Cullen P.J., Steinberg F. (2018). To degrade or not to degrade: Mechanisms and significance of endocytic recycling. Nat. Rev. Mol. Cell Biol..

[20] Markmann S., Krambeck S., Hughes C.J., Mirzaian M., Aerts J.M.F.G., Saftig P., Schweizer M., Vissers J.P.C., Braulke T., Damme M. (2017). Quantitative proteome analysis of mouse liver lysosomes provides evidence for mannose 6-phosphate-independent targeting mechanisms of acid hydrolases in mucolipidosis II. Mol. Cell. Proteom..

[21] Reczek D., Schwake M., Schröder J., Hughes H., Blanz J., Jin X., Brondyk W., Van Patten S., Edmunds T., Saftig P. (2007). LIMP-2 is a receptor for lysosomal mannose-6-phosphate-independent targeting of beta-glucocerebrosidase. Cell.

[22] Huotari J., Helenius A. (2011). Endosome maturation. EMBO J..

[23] Wandinger-Ness A., Zerial M. (2014). Rab Proteins and the Compartmentalization of the Endosomal System. Cold Spring Harb. Perspect. Biol..

[24] Saffi G.T., Botelho R.J. (2019). Lysosome Fission: Planning for an Exit. Trends Cell Biol..

[25] Miaczynska M., Stenmark H. (2008). Mechanisms and functions of endocytosis. J. Cell Biol..

[26] Kaushik S., Cuervo A.M. (2012). Chaperone-mediated autophagy: A unique way to enter the lysosome world. Trends Cell Biol..

[27] Schuck S. (2020). Microautophagy—Distinct molecular mechanisms handle cargoes of many sizes. J. Cell Sci..

[28] Tekirdag K., Cuervo A.M. (2018). Chaperone-mediated autophagy and endosomal microautophagy: Jointed by a chaperone. J. Biol. Chem..

[29] Klionsky D.J., Codogno P. (2013). The Mechanism and Physiological Function of Macroautophagy. J. Innate Immun..

[30] Napolitano G., Ballabio A. (2016). TFEB at a glance. J. Cell Sci..

[31] Garcia D., Shaw R.J. (2017). AMPK: Mechanisms of cellular energy sensing and restoration of metabolic balance. Mol. Cell.

[32] Carroll B., Dunlop E.A. (2017). The lysosome: A crucial hub for AMPK and mTORC1 signaling. Biochem. J..

[33] Saxton R.A., Sabatini D.M. (2017). mTOR Signaling in Growth, Metabolism, and Disease. Cell.

[34] Liu G.Y., Sabatini D.M. (2020). mTOR at the nexus of nutrition, growth, ageing and disease. Nat. Rev. Mol. Cell Biol..

[35] Inpanathan S., Botelho R.J. (2019). The Lysosome Signaling Platform: Adapting With the Times. Front. Cell Dev. Biol..

[36] Settembre C., Fraldi A., Medina D.L., Ballabio A. (2013). Signals from the lysosome: A control centre for cellular clearance and energy metabolism. Nat. Rev. Mol. Cell Biol..

[37] Colaço A., Jäättelä M. (2017). Ragulator—A multifaceted regulator of lysosomal signaling and trafficking. J. Cell Biol..

[38] Wang S., Tsun Z.Y., Wolfson R., Shen K., Wyant G.A., Plovanich M.E., Yuan E.D., Jones T.D., Chantranupong L., Comb W. (2015). The amino acid transporter SLC38A9 is a key component of a lysosomal membrane complex that signals arginine sufficiency to mTORC1. Science.

[39] Lawrence R.E., Zoncu R. (2019). The lysosome as a cellular centre for signaling, metabolism and quality control. Nat. Cell Biol..

[40] Lawrence R.E., Cho K.F., Rappold R., Thrun A., Tofaute M., Kim D.J., Moldavski O., Hurley J.H., Zoncu R. (2018). A nutrient-induced affinity switch controls mTORC1 activation by its Rag GTPase–Ragulator lysosomal scaffold. Nat. Cell Biol..

[41] Settembre C., Ballabio A. (2014). Lysosomal adaptation: How the lysosome responds to external cues. Cold Spring Harb. Perspect. Biol..

[42] Zhang C.S., Bin J., Li M., Zhu M., Peng Y., Zhang Y.L., Wu Y.Q., Li T.Y., Liang Y., Lu Z. (2014). The Lysosomal v-ATPase-Ragulator Complex Is a Common Activator for AMPK and mTORC1, Acting as a Switch between Catabolism and Anabolism. Cell Metab..

[43] Zhang C.S., Hawley S.A., Zong Y., Li M., Wang Z., Gray A., Ma T., Cui J., Feng J.W., Zhu M. (2017). Fructose-1,6-bisphosphate and aldolase mediate glucose sensing by AMPK. Nature.

[44] Sardiello M. (2016). Transcription factor EB: From master coordinator of lysosomal pathways to candidate therapeutic target in degenerative storage diseases. Ann. N. Y. Acad. Sci..

[45] Palmieri M., Impey S., Kang H., di Ronza A., Pelz K., Sardiello M., Ballabio A. (2011). Characterization of the CLEAR network reveals an integrated control of cellular clearance pathways. Hum. Mol. Genet..

[46] Garrity A.G., Wang W., Collier C.M.D., Levey S.A., Gao Q., Xu H. (2016). The endoplasmic reticulum, not the pH gradient, drives calcium refilling of lysosomes. Elife.

[47] Cheng X., Shen D., Samie M., Xu H. (2010). Mucolipins: Intracellular TRPML1-3 Channels. FEBS Lett..

[48] Patel S., Marchant J.S., Brailoiu E. (2010). Two-pore channels: Regulation by NAADP and customized roles in triggering calcium signals. Cell Calcium.

[49] Wang W., Gao Q., Yang M., Zhang X., Yu L., Lawas M., Li X., Bryant-Genevier M., Southall N.T., Marugan J. (2015). Up-regulation of lysosomal TRPML1 channels is essential for lysosomal adaptation to nutrient starvation. Proc. Nat. Acad. Sci. USA.

[50] Medina D.L., Di Paola S., Peluso I., Armani A., De Stefani D., Venditti R., Montefusco S., Scotto-Rosato A., Prezioso C., Forrester A. (2015). Lysosomal calcium signaling regulates autophagy through calcineurin and TFEB. Nat. Cell Biol..

[51] Sun X., Yang Y., Zhong X.Z., Cao Q., Zhu X.H., Zhu X., Dong X.P. (2018). A negative feedback regulation of MTORC1 activity by the lysosomal Ca^2+^ channel MCOLN1 (mucolipin 1) using a CALM (calmodulin)-dependent mechanism. Autophagy.

[52] Pu J., Guardia C.M., Keren-Kaplan T., Bonifacino J.S. (2016). Mechanisms and functions of lysosome positioning. J. Cell Sci..

[53] Buratta S., Tancini B., Sagini K., Delo F., Chiaradia E., Urbanelli L., Emiliani C. (2020). Lysosomal Exocytosis, Exosome Release and Secretory Autophagy: The Autophagic- and Endo-Lysosomal Systems Go Extracellular. Int. J. Mol. Sci..

[54] Samie M.A., Xu H. (2014). Lysosomal exocytosis and lipid storage disorders. J. Lipid Res..

[55] Medina D.L., Fraldi A., Bouche V., Annunziata F., Mansueto G., Spampanato C., Puri C., Pignata A., Martina J.A., Sardiello M. (2011). Transcriptional activation of lysosomal exocytosis promotes cellular clearance. Dev. Cell.

[56] Samie M., Wang X., Zhang X., Goschka A., Li X., Cheng X., Gregg E., Azar M., Zhuo Y., Garrity A.G. (2013). A TRP channel in the lysosome regulates large particle phagocytosis via focal exocytosis. Dev. Cell.

[57] Andrews N.W. (2017). Detection of Lysosomal Exocytosis by Surface Exposure of Lamp1 Luminal Epitopes. Methods Mol. Biol..

[58] Marques A.R.A., Saftig P. (2019). Lysosomal storage disorders—Challenges, concepts and avenues for therapy: Beyond rare diseases. J. Cell Sci..

[59] Bonifacino J.S., Rojas R. (2006). Retrograde transport from endosomes to the trans-Golgi network. Nat. Rev. Mol. Cell Biol..

[60] Jaiswal J.K., Andrews N.W., Simon S.N. (2002). Membrane proximal lysosomes are the major vesicles responsible for calcium-dependent exocytosis in non secretory cells. J. Cell Biol..

[61] Knabbe J., Nassal J.P., Verhage M., Kuner T. (2018). Secretory vesicle trafficking in awake and anaesthetized mice: Differential speeds in axons versus synapses. J. Physiol..

[62] Bandyopadhyay D., Cyphersmith A., Zapata J.A., Kim Y.J., Payne C.K. (2014). Lysosome Transport as a Function of Lysosome Diameter. PLoS ONE.

[63] Cross J.A., Dodding M.P. (2019). Motor–cargo adaptors at the organelle–cytoskeleton interface. Curr. Opin. Cell Biol..

[64] De Pace R., Britt D.J., Mercurio J., Foster A.M., Djavaherian L., Hoffmann V., Abebe D., Bonifacino J.S. (2020). Synaptic vesicle precursors and lysosomes are transported by different mechanisms in the axon of mammalian neurons. Cell Rep..

[65] Hirokawa N., Noda Y., Tanaka Y., Niwa S. (2009). Kinesin superfamily motor proteins and intracellular transport. Nat. Rev. Mol. Cell Biol..

[66] Sanger A., Yip Y.Y., Randall T.S., Pernigo S., Steiner R.A., Dodding M.P. (2017). SKIP controls lysosome positioning using a composite kinesin-1 heavy and light chain-binding domain. J. Cell Sci..

[67] Loubéry S., Wilhelm C., Hurbain I., Neveu S., Louvard D., Coudrier E. (2008). Different Microtubule Motors Move Early and Late Endocytic Compartments. Traffic.

[68] Bentley M., Decker H., Luisi J., Banker G. (2015). A novel assay reveals preferential binding between Rabs, kinesins, and specific endosomal subpopulations. J. Cell Biol..

[69] Marx A., Hoenger A., Mandelkow E. (2009). Structures of Kinesin Motor Proteins. Cell Motil. Cytoskel..

[70] Cardoso C.M.P., Groth-Pedersen L., Høyer-Hansen M., Kirkegaard T., Corcelle E., Andersen J.S., Jäättelä M., Nylandsted J. (2009). Depletion of Kinesin 5B Affects Lysosomal Distribution and Stability and Induces Peri-Nuclear Accumulation of Autophagosomes in Cancer Cells. PLoS ONE.

[71] Khatter D., Sindhwani A., Sharma M. (2015). Arf-like GTPase Arl8: Moving from the periphery to the center of lysosomal biology. Cell Logist..

[72] Rosa-Ferreira C., Munro S. (2011). Arl8 and SKIP act together to link lysosomes to kinesin-1. Dev. Cell.

[73] Starcevic M., Dell’Angelica E.C. (2004). Identification of snapin and three novel proteins (BLOS1, BLOS2, and BLOS3/reduced pigmentation) as subunits of biogenesis of lysosome-related organelles complex-1 (BLOC-1). J. Biol. Chem..

[74] Pu J., Schindler C., Jia R., Jarnik M., Backlund P., Bonifacino J.S. (2015). BORC, a multisubunit complex that regulates lysosome positioning. Dev. Cell.

[75] Balderhaar H.J., Ungermann C. (2013). CORVET and HOPS tethering complexes—Coordinators of endosome and lysosome fusion. J. Cell Sci..

[76] Pankiv S., Alemu E.A., Brech A., Bruun J.A., Lamark T., Øvervatn A., Bjørkøy G., Johansen T. (2010). FYCO1 is a Rab7 effector that binds to LC3 and PI3P to mediate microtubule plus end–directed vesicle transport. J. Cell Biol..

[77] Raiborg C., Stenmark H. (2016). Plasma membrane repairs by small GTPase Rab3a. J. Cell Biol..

[78] Jordens I., Fernandez-Borja M., Marsman M., Dusseljee S., Janssen L., Calafat J., Janssen H., Wubbolts R., Neefjes J. (2001). The Rab7 effector protein RILP controls lysosomal transport by inducing the recruitment of dynein-dynactin motors. Curr. Biol..

[79] Willett R., Martina J.A., Zewe J.P., Wills R., Hammond G.R.V., Puertollano R. (2017). TFEB regulates lysosomal positioning by modulating TMEM55B expression and JIP4 recruitment to lysosomes. Nat. Commun..

[80] Li X., Rydzewski N., Hider A., Zhang X., Yang J., Wang W., Gao Q., Cheng X., Xu H. (2016). A molecular mechanism to regulate lysosome motility for lysosome positioning and tubulation. Nat. Cell Biol..

[81] Carnell M., Zech T., Calaminus S.D., Ura S., Hagedorn M., Johnston S.A., May R.C., Soldati T., Machesky L.M., Insall R.H. (2011). Actin polymerization driven by WASH causes V-ATPase retrieval and vesicle neutralization before exocytosis. J. Cell Biol..

[82] Derivery E., Helfer E., Henriot V., Gautreau A. (2012). Actin Polymerization Controls the Organization of WASH Domains at the Surface of Endosomes. PLoS ONE.

[83] Monfregola I., Napolitano G., D’Urso M., Lappalainen P., Ursini M.V. (2010). Functional Characterization of Wiskott-Aldrich Syndrome Protein and Scar Homolog (WASH), a Bi-modular Nucleation-promoting Factor Able to Interact with Biogenesis of Lysosome-related Organelle Subunit 2 (BLOS2) and γ-Tubulin. J. Biol. Chem..

[84] Encarnação M., Espada L., Escrevente C., Mateus D., Ramalho J., Michelet X., Santarino I., Hsu V.W., Brenner M.B., Barral D.C. (2016). A Rab3a-dependent complex essential for lysosome positioning and plasma membrane repair. J. Cell Biol..

[85] Van Bommel B., Konietzny A., Kobler O., Bär J., Mikhaylova M. (2019). F-actin patches associated with glutamatergic synapses control positioning of dendritic lysosomes. EMBO J..

[86] Nakamura S., Yoshimori T. (2017). New insights into autophagosome–lysosome fusion. J. Cell Sci..

[87] Karlovich Lund V., Lindegaard Madsen K., Kjaerulff O. (2018). Drosophila Rab2 controls endosome-lysosome fusion and LAMP delivery to late endosomes. Autophagy.

[88] Han J., Pluhackova K., Böckmann R.A. (2017). The Multifaceted Role of SNARE Proteins in Membrane Fusion. Front. Physiol..

[89] Rao S.K., Huynh C., Proux-Gillardeaux V., Galli T., Andrews N.W. (2004). Identification of SNAREs involved in synaptotagmin VII-regulated lysosomal exocytosis. J. Biol. Chem..

[90] Lopez Sanjurjo C.I., Tovey S.C., Taylor C. (2014). Rapid Recycling of Ca^2+^ Between IP_3_-Sensitive Stores and Lysosomes. PLoS ONE.

[91] Más Gómez N., Lu W., Lim J.C., Kiselyov K., Campagno K.E., Grishchuk Y., Slaugenhaupt S.A., Pfeffer B.A., Fliesler S.J., Mitchell C.H. (2018). Robust lysosomal calcium signaling through channel TRPML1 is impaired by lysosomal lipid accumulation. FASEB J..

[92] Czibener C., Sherer N.M., Becker S.M., Pypaert M., Hui E., Chapman E.R., Mothes W., Andrews N.W. (2006). Ca^2+^ and synaptotagmin VII–dependent delivery of lysosomal membrane to nascent phagosomes. J. Cell Biol..

[93] MacDougall D.D., Lin Z., Chon N.L., Jackman S.L., Lin H., Knight J.D., Anantharam A. (2018). The high-affinity calcium sensor synaptotagmin-7 serves multiple roles in regulated exocytosis. J. Gen. Physiol..

[94] Di Paola S., Scotto-Rosato A., Medina D.L. (2018). TRPML1: The Ca^(2+)^ retaker of the lysosome. Cell Calcium.

[95] Cao Q., Yang Y., Zhong X.Z., Dong X.P. (2017). The lysosomal Ca^2+^ release channel TRPML1 regulates lysosome size by activating calmodulin. J. Biol. Chem..

[96] Xu J., Toops K.A., Diaz F., Carvajal-Gonzalez J.M., Gravotta D., Mazzoni F., Schreiner R., Rodriguez-Boulan E., Lakkaraju A. (2012). Mechanism of polarized lysosome exocytosis in epithelial cells. J. Cell Sci..

[97] Korolchuk V.I., Saiki S., Lichtenberg M., Siddiqi F.H., Roberts E.A., Imarisio S., Rubinsztein D.C. (2011). Lysosomal positioning coordinates cellular nutrient responses. Nat. Cell Biol..

[98] Pu J., Keren-Kaplan T., Bonifacino J.S. (2017). A Ragulator–BORC interaction controls lysosome positioning in response to amino acid availability. J. Cell Biol..

[99] Filipek P.A., de Araujo M.E.G., Vogel G.F., De Smet C.H., Eberharter D., Rebsamen M., Rudashevskaya E.L., Kremser L., Yordanov T., Tschaikner P. (2017). LAMTOR/Ragulator is a negative regulator of Arl8b- and BORC-dependent late endosomal positioning. J. Cell Biol..

[100] Rocha N., Kuijl C., van der Kant R., Janssen L., Houben D., Janssen H., Zwart W., Neefjes J. (2009). Cholesterol sensor ORP1L contacts the ER protein VAP to control Rab7-RILP-p150 Glued and late endosome positioning. J. Cell Biol..

[101] Lang T., Bruns D., Wenzel D., Riedel D., Holroyd P., Thiele C., Jahn R. (2001). SNAREs are concentrated in cholesterol-dependent clusters that define docking and fusion sites for exocytosis. EMBO J..

[102] Fraldi A., Annunziata F., Lombardi A., Kaiser H.J., Medina D.L., Spampanato C., Fedele A.O., Polishchuk R., Sorrentino N.C., Simons K. (2010). Lysosomal fusion and SNARE function are impaired by cholesterol accumulation in lysosomal storage disorders. EMBO J..

[103] Weiss N. (2012). Cross-talk between TRPML1 channel, lipids and lysosomal storage diseases. Commun. Integr. Biol..

[104] Yogalingam G., Bonten E.J., van de Vlekkert D., Hu H., Moshiach S., Connell S.A., D’Azzo A. (2008). Neuraminidase 1 is a negative regulator of lysosomal exocytosis. Dev. Cell.

[105] Tam C., Idone V., Devlin C., Fernandes M.C., Flannery A., He X., Schuchman E., Tabas I., Andrews N.W. (2010). Exocytosis of acid sphingomyelinase by wounded cells promotes endocytosis and plasma membrane repair. J. Cell Biol..

[106] Nair S.V., Narendradev N.D., Nambiar R.P., Kumar R., Srinivasula S.M. (2020). Naturally occurring and tumor-associated variants of RNF167 promote lysosomal exocytosis and plasma membrane resealing. J. Cell Sci..

[107] Heuser J. (1989). Changes in lysosome shape and distribution correlated with changes in cytoplasmic pH. J. Cell Biol..

[108] Glunde K., Guggino S.E., Solaiyappan M., Pathak A.P., Ichikawa Y., Bhujwalla Z.M. (2003). Extracellular Acidification Alters Lysosomal Trafficking in Human Breast Cancer Cells. Neoplasia.

[109] Sundler R. (1997). Lysosomal and cytosolic pH as regulators of exocytosis in mouse macrophages. Acta Physiol. Scand..

[110] Steffan J.J., Snider J.L., Skalli O., Welbourne T., Cardelli J.A. (2009). Na+/H+ exchangers and RhoA regulate acidic extracellular pH-induced lysosome trafficking in prostate cancer cells. Traffic.

[111] Miao Y., Li G., Zhang X., Xu H., Abraham S.N. (2015). A TRP Channel Senses Lysosome Neutralization by Pathogens to Trigger Their Expulsion. Cell.

[112] Zhitomirsky B., Assaraf Y.G. (2017). Lysosomal accumulation of anticancer drugs triggers lysosomal exocytosis. Oncotarget.

[113] Buratta S., Urbanelli L., Ferrara G., Sagini K., Goracci L., Emiliani C. (2015). A role for the autophagy regulator Transcription Factor EB in amiodarone-induced phospholipidosis. Biochem. Pharmacol..

[114] Geisslinger F., Müller M., Vollmar A.M., Bartel K. (2020). Targeting Lysosomes in Cancer as Promising Strategy to Overcome Chemoresistance—A Mini Review. Front. Oncol..

[115] Padamsey Z., McGuinness L., Bardo S.J., Reinhart M., Tong R., Hedegaard A., Hart M.L., Emptage N.J. (2017). Activity-Dependent Exocytosis of Lysosomes Regulates the Structural Plasticity of Dendritic Spines. Neuron.

[116] Huynh C., Roth D., Ward D.M., Kaplan J., Andrews N.W. (2004). Defective lysosomal exocytosis and plasma membrane repair in Chediak Higashi/beige cells. Proc. Natl. Acad. Sci. USA.

[117] Arantes R.M.E., Andrews N.W. (2006). A Role for Synaptotagmin VII-Regulated Exocytosis of Lysosomes in Neurite Outgrowth from Primary Sympathetic Neurons. J. Neurosci..

[118] Obino D., Diaz J., Sáez J.S., Ibañez-Vega J., Sáez P.J., Alamo M., Lankar D., Yuseff M.I. (2017). Vamp-7–dependent secretion at the immune synapse regulates antigen extraction and presentation in B-lymphocytes. Mol. Biol. Cell.

[119] Zhao H., Ito Y., Chappel J., Andrews N.W., Teitelbaum S.L., Ross F.P. (2008). Synaptotagmin VII Regulates Bone Remodeling by Modulating Osteoclast and Osteoblast Secretion. Dev. Cell.

[120] Jung J., Jo H.W., Kwon H., Jeong N.Y. (2014). ATP Release through Lysosomal Exocytosis from Peripheral Nerves: The Effect of Lysosomal Exocytosis on Peripheral Nerve Degeneration and Regeneration after Nerve Injury. Biomed. Res. Int..

[121] Rodríguez A., Webster P., Ortego J., Andrews N.W. (1997). Lysosomes behave as Ca^2+^-regulated exocytic vesicles in fibroblasts and epithelial cells. J. Cell Biol..

[122] Rodríguez A., Martinez I., Chung A., Berlot C.H., Andrews N.W. (1999). cAMP regulates Ca^2+^-dependent exocytosis of lysosomes and lysosome-mediated cell invasion by trypanosomes. J. Biol Chem..

[123] Reddy A., Caler E.V., Andrews N.W. (2001). Plasma membrane repair is mediated by Ca^(2+)^-regulated exocytosis of lysosomes. Cell.

[124] Castro-Gomes T., Corrotte M., Tam C., Andrews N.W. (2016). Plasma Membrane Repair Is Regulated Extracellularly by Proteases Released from Lysosomes. PLoS ONE.

[125] Underhill D.M., Goodridge H.S. (2012). Information processing during phagocytosis. Nat. Rev. Immunol..

[126] Münz C. (2017). The Autophagic Machinery in Viral Exocytosis. Front. Microbiol..

[127] Urbanelli L., Buratta S., Tancini B., Sagini K., Delo F., Porcellati S., Emiliani C. (2019). The Role of Extracellular Vesicles in Viral Infection and Transmission. Vaccines.

[128] Ghosh S., Dellibovi-Ragheb T.A., Kerviel A., Pak E., Qiu Q., Fisher M., Takvorian P.M., Bleck C., Hsu V.W., Fehr A.R. (2020). β-Coronaviruses Use Lysosomes for Egress Instead of the Biosynthetic Secretory Pathway. Cell.

[129] Martinez I., Chakrabarti S., Hellevik T., Morehead J., Fowler K., Andrews N.W. (2000). Synaptotagmin VII Regulates Ca^2+^-Dependent Exocytosis of Lysosomes in Fibroblasts. J. Cell Biol..

[130] Chen G., Zhang Z., Wei Z., Cheng Q., Li X., Li W., Duan S., Gu X. (2012). Lysosomal exocytosis in Schwann cells contributes to axon remyelination. Glia.

[131] Lohmer L.L., Kelley L.C., Hagedorn E.J., Sherwood D.R. (2014). Invadopodia and basement membrane invasion in vivo. Cell Adh Migr..

[132] Naegeli K.M., Hastie E., Garde A., Wang Z., Keeley D.P., Gordon K.L., Pani A.M., Kelley L.C., Morrissey M.A., Chi Q. (2017). Cell invasion in vivo via rapid exocytosis of a transient lysosome-derived membrane domain. Dev. Cell.

[133] Damaghi M., Tafreshi N.K., Lloyd M.C., Sprung R., Estrella V., Wojtkowiak J.W., Morse D.L., Koomen J.M., Bui M.M., Gatenby R.A. (2015). Chronic acidosis in the tumour microenvironment selects for overexpression of LAMP2 in the plasma membrane. Nat. Commun..

[134] Funato Y., Yoshida A., Hirata Y., Hashizume O., Yamazaki D., Miki H. (2020). The Oncogenic PRL Protein Causes Acid Addiction of Cells by Stimulating Lysosomal Exocytosis. Dev. Cell.

[135] Sobacchi C., Schulz A., Coxon F.P., Villa A., Helfrich M.H. (2013). Osteopetrosis: Genetics, treatment and new insights into osteoclast function. Nat. Rev. Endocrinol..

[136] Lacombe J., Karsenty G., Ferron M. (2013). Regulation of lysosome biogenesis and functions in osteoclasts. Cell Cycle.

[137] Ireton K., Van Ngo H., Bhalla M. (2018). Interaction of microbial pathogens with host exocytic pathways. Cell. Microbiol..

[138] Roche P.A., Furuta K. (2015). The ins and outs of MHC class II-mediated antigen processing and presentation. Nat. Rev. Immunol..

[139] Dou Y., Wu H.J., Li H.Q., Qin S., Wang Y.E., Li J., Lou H.F., Chen Z., Li X.M., Luo Q.M. (2012). Microglial migration mediated by ATP-induced ATP release from lysosomes. Cell Res..

[140] Huang P., Zou Y., Zhong X.Z., Cao Q., Zhao K., Zhu M.X., Murrell-Lagnado R., Dong X.P. (2014). P2X4 forms functional ATP-activated cation channels on lysosomal membranes regulated by luminal pH. J. Biol. Chem..

[141] Xiong Y., Sun S., Teng S., Jin M., Zhou Z. (2018). Ca^2+^-Dependent and Ca^2+^-Independent ATP Release in Astrocytes. Front. Mol. Neurosci..

[142] Datta G., Miller N.M., Afghah Z., Geiger J.D., Chen X. (2019). HIV-1 gp120 Promotes Lysosomal Exocytosis in Human Schwann Cells. Front. Cell Neurosci..

[143] Machado E., White-Gilbertson S., van de Vlekkert D., Janke L., Moshiach S., Campos Y., Finkelstein D., Gomero E., Mosca R., Qiu X. (2015). Regulated lysosomal exocytosis mediates cancer progression. Sci. Adv..

[144] Kundu S.T., Grzeskowiak C.L., Fradette J.J., Gibson L.A., Rodriguez L.B., Creighton C.J., Scott K.L., Gibbons D.L. (2018). TMEM106B drives lung cancer metastasis by inducing TFEB-dependent lysosome synthesis and secretion of cathepsins. Nat. Commun..

[145] Ballabio A., Bonifacino J.S. (2020). Lysosomes as dynamic regulators of cell and organismal homeostasis. Nat. Rev. Mol. Cell Biol..

[146] Polishchuk E.V., Concilli M., Iacobacci S., Chesi G., Pastore N., Piccolo P., Paladino S., Baldantoni D., van IJzendoorn S.C.D., Chan J. (2014). Wilson Disease Protein ATP7B Utilizes Lysosomal Exocytosis to Maintain Copper Homeostasis. Dev. Cell..

[147] Kukic I., Kelleher S.L., Kiselyov K. (2014). Zn^2+^ efflux through lysosomal exocytosis prevents Zn2+-induced toxicity. J. Cell Sci..

[148] Cui W., Sathyanarayan A., Lopresti M., Aghajan M., Chen C., Mashek D.G. (2020). Lipophagy-derived fatty acids undergo extracellular efflux via lysosomal exocytosis. Autophagy.

[149] Malik B.R., Maddison D.C., Smith G.A., Peters O.M. (2019). Autophagic and endo-lysosomal dysfunction in neurodegenerative disease. Mol. Brain.

[150] Bonam S.R., Wang F., Muller S. (2019). Lysosomes as a therapeutic target. Nat. Rev. Drug Discov..

[151] Klein D., Büssow H., Ngamli Fewou S., Gieselmann V. (2005). Exocytosis of storage material in a lysosomal disorder. Biochem. Biophys. Res. Commun..

[152] Spampanato C., Feeney E., Li L., Cardone M., Lim J.A., Annunziata F., Zare H., Polishchuk R., Puertollano R., Parenti G. (2013). Transcription factor EB (TFEB) is a new therapeutic target for Pompe disease. EMBO Mol. Med..

[153] Palmieri M., Pal R., Nelvagal H.R., Lotfi P., Stinnett G.R., Seymour M.L., Chaudhury A., Bajaj L., Bondar V.V., Bremner L. (2017). mTORC1-independent TFEB activation via Akt inhibition promotes cellular clearance in neurodegenerative storage diseases. Nat. Commun..

[154] Contreras P.S., Tapia P.J., González-Hódar L., Peluso I., Soldati C., Napolitano G., Matarese M., Las Heras M., Valls C., Martinez A. (2020). c-Abl inhibition activates TFEB and promotes cellular clearance in a lysosomal disorder. Iscience.

[155] Shen D., Wang X., Li X., Zhang X., Yao Z., Dibble S., Dong X., Yu T., Lieberman A.P., Showalter H.D. (2012). Lipid storage disorders block lysosomal trafficking by inhibiting a TRP channel and lysosomal calcium release. Nat. Commun..

[156] Feng X., Xiong J., Lu Y., Xia X., Zhu M.X. (2014). Differential mechanisms of action of the mucolipin synthetic agonist, ML-SA1, on insect TRPML and mammalian TRPML1. Cell Calcium.

[157] Xu Y., Du S., Marsh J.A., Horie K., Sato C., Ballabio A., Karch C.M., Holtzman D.M., Zheng H. (2020). TFEB regulates lysosomal exocytosis of tau and its loss of function exacerbates tau pathology and spreading. Mol. Psychiatry.

[158] Tsunemi T., Perez-Rosello T., Ishiguro Y., Yoroisaka A., Jeon S., Hamada K., Rammonhan M., Wong Y.C., Xie Z., Akamatsu W. (2019). Increased Lysosomal Exocytosis Induced by Lysosomal Ca^2+^ Channel Agonists Protects Human Dopaminergic Neurons from α-Synuclein Toxicity. J. Neurosci..

[159] Bae M., Patel N., Xu H., Lee M., Tominaga-Yamanaka K., Nath A., Geiger J., Gorospe M., Mattson M.P., Haughey N.J. (2014). Activation of TRPML1 clears intraneuronal abeta in preclinical models of HIV infection. J. Neurosci..

[160] Van de Vlekkert D., Demmers J., Nguyen X., Campos Y., Machado E., Annunziata I., Hu H., Gomero E., Qiu X., Bongiovanni A. (2019). Excessive exosome release is the pathogenic pathway linking a lysosomal deficiency to generalized fibrosis. Sci. Adv..

